# Host Molecule Incorporation into HIV Virions, Potential Influences in HIV Pathogenesis

**DOI:** 10.3390/v14112523

**Published:** 2022-11-14

**Authors:** Olivia Munoz, Riddhima Banga, Matthieu Perreau

**Affiliations:** Division of Immunology and Allergy, Lausanne University Hospital, University of Lausanne, 1011 Lausanne, Switzerland

**Keywords:** HIV, replication cycle, protein incorporation, pathogenesis

## Abstract

During the last phase of HIV viral production, nascent HIV virions acquire a fraction of the cellular lipid membrane to create the external lipid envelope, a process by which cellular proteins present on the surface of the infected cell can be incorporated along with Env trimers. Interestingly, several studies indicated that these incorporated host molecules could conserve their biological activity and consequently contribute to HIV pathogenesis either by enhancing the infectivity of HIV virions, their tissue tropism or by affecting immune cell functions. The following review will describe the main approaches used to characterize membrane bound host molecule incorporation into HIV virions, the proposed mechanisms involved, and the role of a non-exhaustive list of incorporated molecules.

## 1. Introduction

Since the beginning of the epidemic, more than 84.2 million individuals have been infected by HIV. In 2021, about 38.4 million people have already died from HIV-related diseases (https://www.who.int/gho/hiv/en/) (accessed on 15 August 2022) and 37.6 million people were living with HIV infection, but none of them were able to naturally clear the infection. Indeed, in the absence of treatment, 99% of HIV-infected people experience a gradual decline in their CD4 T cell count. When the CD4 T cell count drops to around 200 cells/mm^3^ of blood, these individuals enter the final stage of the infection, the acquired immunodeficiency syndrome (AIDS), eight to ten years after infection [1,2,3]. At that stage, the immune system collapses and is no longer able to protect individuals from opportunistic pathogens, and HIV-infected individuals ultimately die from infections or cancers [1,4].

The reasons for this extremely efficient way for HIV to resist to the pressure exerted by the immune system probably reside in complex immuno-virological mechanisms [5,6,7,8,9]. The functional impairment of HIV-specific CD4 and CD8 T cells (reviewed in Fenwick et al., 2019 [7]), the difficulty in generating broadly neutralizing antibodies by HIV-specific B cells (reviewed in Havenar-Daughton et al., 2017 [8]), as well as the ability of the virus to establish a viral reservoir capable of remaining in a latent state, and therefore invisible to the immune system (reviewed in Cohn et al., 2020 [9]) contribute to the persistence of the virus [7,8,9]. 

Since HIV is an enveloped virus, HIV virions can incorporate host molecules located either at the membrane or at the cytoplasmic levels during the production process [10,11,12,13,14,15,16]. Interestingly, several studies indicated that these incorporated molecules could still harbor biological activities [17,18,19]. 

A mathematical model estimated that between 100 million and 100 billion viruses could be produced and eliminated from the body of an HIV-infected individual every day [20]. Due to the extent of viruses produced and the capacity of HIV virions to incorporate host molecules during the budding phase, several studies focused on their potential impact during HIV pathogenesis. The present review therefore proposed to focus on the main experimental approaches used to identify membrane bound host molecules incorporated into HIV virions, the mechanisms involved in the incorporation process and the potential role of selected incorporated molecules in HIV pathogenesis.

## 2. Experimental Strategies to Characterize Host Proteins Incorporation into HIV Virions

**Figure 1:**
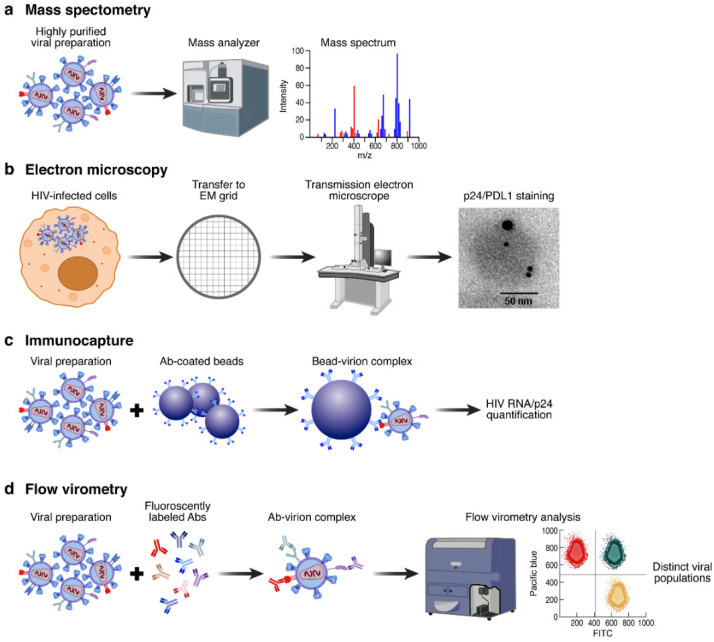
Methods to characterize host protein incorporation into HIV virions: (**a**) mass spectrometry is the gold standard to deeply characterize protein compositions of viral preparations; (**b**) electron microscopy and more recently the use of immuno-gold labeling are becoming standard tools to characterize host protein interaction/incorporation in HIV virions; (**c**) the immuno-capture of HIV virions is a two-step process; first, the capture using beads or plates coated with mAbs and second, the detection of either HIV RNA or p24 to quantify HIV virions; and (**d**) flow virometry represents a promising tool, where HIV virions could be detected and characterized using fluorescently labeled mAbs.

The deep structural characterization of viruses has, for a long time, been limited by their relatively small size. However, in the last decades, the development of novel technological tools allowed for a better characterization of HIV virion structures as well as their protein compositions. Because each technique has its advantages and disadvantages; e.g., the sensitivity for mass spectrometry, the robustness for western blot and electron microscopy, and the small amount of required protein for immune capture (Figure 1), most recent studies proposed combined approaches to characterize the host protein spectra of HIV virions and their potential factors during HIV pathogenesis [21,22]. The following section will focus on the characteristics, advantages, and limitations of the major experimental strategies used to characterize HIV virion envelope composition.

Mass spectrometry (MS) (Figure 1a) or coupling methods such as liquid chromatography–MS (LC-MS) are considered gold standards for the characterization of the protein composition of several viral sources [22,23,24]. Indeed, the high sensitivity in determining the protein quantity and composition of several viruses has improved the understanding of the viral structure and host protein interactions [21,22,24]. In addition, MS is actually the only experimental strategy that does not require prior knowledge of a virus-incorporated host factor [22]. However, this technique has mostly been used on HIV virions produced in vitro from cell lines and not on HIV virions collected directly from ex vivo samples from body fluid studies [25]. Even though MS has been a pioneer tool in the identification of host molecules incorporated into HIV virions, no insights into the host protein profile of a single virion or the co-incorporation of host molecules or the precise localization into the lipid envelope have been obtained, as the analyses were performed on bulk viral preparations. 

Over the past decades, electron microscopy (Figure 1b) became a powerful approach for the characterization of the structural organization of HIV virions. While mature HIV virions could be easily recognized by the specific conical shape of the capsid using electron microscopy, the characterization of host protein incorporation into the HIV virion lipid envelope requires the use of a specific immunogold-labeling [26,27]. Immunogold staining of cellular sections infected with viruses involves low level of fixation, which may result in the loss of HIV viral structure and limit the visual characterization of HIV virions in these preparations. The development of novel approaches to distinguish several proteins on an individual virion by electron microscopy was therefore developed and illustrated by the recent use of dual immunogold-labeling [19,28]. This approach is based on the use of two primary antibodies (e.g., p24, Env, or host proteins) labeled beads harboring distinct sizes, distinguishable by electron microscopy. This approach classically allows the determination of the co-expression of the two proteins (viral or host proteins) on individual HIV virions [28]. This approach may also provide further information on the localization (e.g., envelope, capsid, matrix) of cellular proteins into HIV virions. 

The immuno-capture assay is a highly specific approach that includes two phases (Figure 1c). The first step consists of the capture of viral particles that can be achieved using either beads or plates coated with antibodies against the proteins of interest. The second step is usually based on the quantification of either p24 or HIV RNA to determine the quantity of viral particles captured by the specific antibodies [12,29,30]. Several studies used this approach to confirm the incorporation of host proteins (e.g., Intercellular Adhesion Molecules-1 (ICAM-1), HLA-DR, and α4β7) [12,29,30]. However, one of the limitations of this technique is the detection of only one protein at a time, preventing the study of the co-incorporation of various host molecules. 

Until recently, the use of flow cytometry to characterize HIV virions was limited due to the difficulties of conventional flow cytometers to detect particles smaller than 400 nm, knowing that the size of HIV virions is comprised between 90–120 nm. However, in the last few years, many efforts were invested in the development of more sensitive instruments and the most recent studies are able to efficiently distinguish individual HIV virion using a new approach called flow virometry (Figure 1d) [31,32,33,34,35]. Most of these studies used fluorescent-labeled in vitro produced HIV virions to facilitate the discrimination between the background noise and viral populations [36,37]. To circumvent the small size of HIV virions and to increase the purity of viral preparations, one study proposed an indirect method by first isolating viral particles using an immuno-capture assay by small magnetic beads (15 nm) coated with gp120 antibodies and second, the HIV virion-bead complex detection using flow cytometry [32]. This study confirmed the presence of two cellular proteins known to be incorporated into HIV virions (i.e., HLA-DR, and LFA-1) and was able to distinguish distinct viral subpopulations based on the co-expression of these two proteins [32]. This method has several advantages; the estimation of the amount of cellular proteins expressed by a single virion as well as the potential co-expression of different proteins, allowing for the differentiation of distinct viral populations based on their host protein incorporation signatures. Indeed, the flow virometry method could represent a considerable advance in virology research and may echo the technological shift that flow cytometry has brought to cellular immunology.

## 3. Mechanisms of Host Protein Incorporation

**Figure 2:**
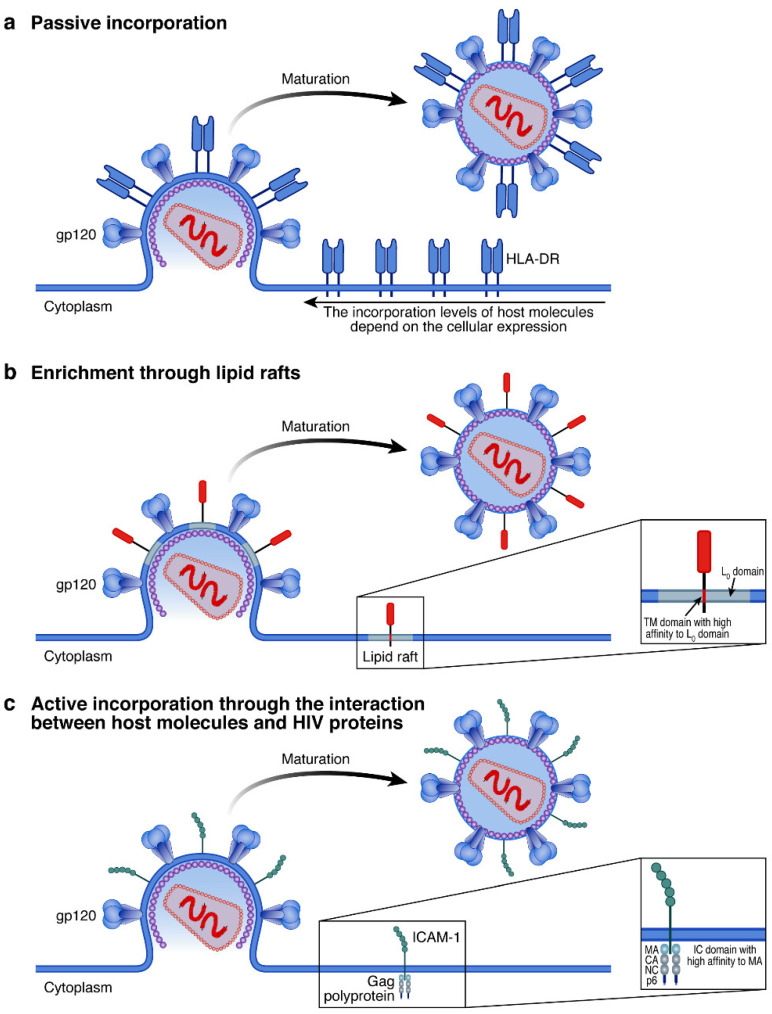
Mechanisms of host protein incorporation into HIV virions: (**a**) passive incorporation; no differences in protein (e.g., HLA-DR in blue) levels between the cellular and viral envelope: and (**b**) lipid composition remodeling (grey fragments) and surface curvature promote the incorporation of specific proteins (e.g., CD59 in red) that have a transmembrane domain with high affinity to the liquid-order phase generated by HIV virions. (**c**) Interaction between HIV proteins (e.g., Gag polyprotein) (violet) and the intracytoplasmic tail of host proteins (e.g., ICAM-1 in green).

Pioneer studies initially proposed that host protein incorporation resulted from a passive mechanism depending on the quantity of proteins expressed on the cell surface and located at close proximity to the budding site (Figure 2a) [38]. 

In agreement with this paradigm, initial findings proposed that during the viral budding process through the lipid rafts, proteins present on the cell surface were passively incorporated into the HIV virion envelope [39,40].

However, more recent studies have demonstrated that the protein levels on HIV-infected cell surfaces did not necessarily correlate with host protein profiles of HIV virions [11,41]. In this context, more recent studies proposed that in addition to a passive mechanism (Figure 2a) and to the enrichment of host molecule incorporation into HIV virions due to the composition of anchored proteins within the lipid raft (Figure 2b), host molecules could also be incorporated via an active process involving direct interactions between host molecules and viral proteins (Figure 2c) [42]. The next section will, therefore, underscore the proposed mechanisms of host protein incorporation and host protein exclusion [41,43]. 

HIV can actively remodel the lipid composition of the cell membrane at the budding site (Figure 2b). The lipid composition remodeling is usually divided into two phases; the first one consists of the formation of a liquid order (L_o_) domain occurring when Gag polyproteins start to oligomerize at the cell membrane [44]. Indeed, Gag polyprotein has a high affinity for Phosphatidylinositol-4,5-bisphosphate (PIP2) that is enriched at the site of assembly and can mediate the clustering of specific GPI-anchored lipid raft within virions envelope to facilitate the incorporation of several proteins (e.g., CD59) [44,45,46]. The second phase is also mediated by Gag and relies on the curvature of the plasma membrane at the assembly site [44]. The enrichment of host proteins with high affinity to the L_o_ phase domain increases protein composition differences and membrane thickness compared to the bulk plasma membrane [44]. Consequently, these changes result in an increase in the line tension, therefore inducing the membrane domains to form circular zones, leading to the formation of budding vesicles enriched in host proteins with high affinity to the L_o_ domain [44,47]. In this context, while the remodeling of the lipid raft at the budding site involves an active mechanism mediated by viral proteins, the incorporation of host molecules might reflect an indirect consequence of this process. 

The selective acquisition of host proteins might also occur as an active process involving the interaction with host molecules and viral proteins (e.g., Gag or Env). Gag polyprotein consists of four fragments: p17 (MA), p24 (CA), NC, and p6. The p17 domain was demonstrated to be crucial for the incorporation of Env, and p17 and NC have been shown to be potential players in protein incorporation [42,48,49,50]. Notably, the incorporation of host molecules (e.g., HLA-DR) has also been shown to be dependent on Env incorporation, even though this observation is still a matter of debate [51,52]. In this context, two independent studies indicated that the interaction of Gag polyprotein with host molecules (i.e., ICAM-1 and PD-L1) promotes their incorporation (Figure 2c) [19,42].

In these cases, the intracytoplasmic tail of host molecules was shown to interact with the matrix (MA) domains of Gag polyprotein thus, driving the active incorporation into HIV Virions [19,42,53]: Of note, the interaction between the host proteins and Gag can remain stable even after the complete maturation of HIV virions. A better understanding and characterization of specific motifs governing the interactions between Gag polyprotein and host molecules would be of great interest to prevent/force the incorporation of host molecules that might have a beneficial/detrimental role during HIV pathogenesis. 

Interestingly, some host proteins (e.g., CD45, CD4, and CD80) can be actively excluded from HIV virion lipid envelopes [41,54,55]. Some authors proposed that this may rely on a mechanism similar to the one used by tetherin, an anti-viral protein known to prohibit HIV virions released during the late phase of the budding process [56]. In this model, the authors proposed that HIV Vpu protein could block the insertion of the GPI domain of tetherin into HIV virion envelope by preventing its co-localization with Gag at the plasma membrane, thus counteracting its potential anti-viral property on mature HIV virions [57]. Whether or not such a mechanism also occurs for other host molecules remains, however, to be determined.

**Figure 3:**
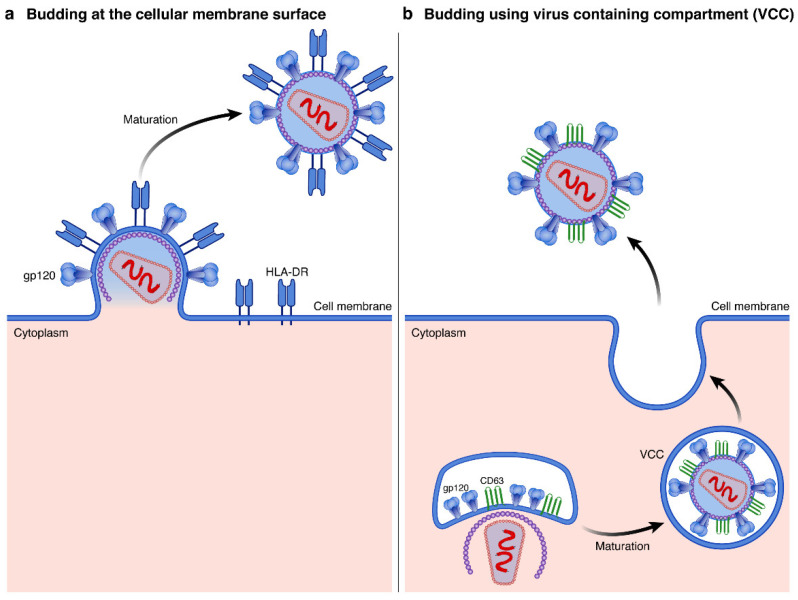
HIV virion assembly and maturation routes: The assembly of HIV virions might occur either (**a**) using the lipid membrane at the cell surface, mostly in lymphoid cells (e.g., HLA-DR in blue) or (**b**) using virus containing compartments, mostly in myeloid cells (e.g., CD63 in green).

HIV virions can use two distinct pathways to bud out from infected cells. HIV virions can bud using the lipid membrane at the surface of cells to create their own envelope (Figure 3a). This mechanism mostly occurs in lymphoid cells but can also happen in myeloid cells [58]. Furthermore, HIV virions can also assemble in virus containing compartment (VCC) in myeloid cells (Figure 3b) [59]. The different production pathways of HIV virions may partially explain the differences in host protein incorporation signatures observed between HIV virions produced in lymphoid vs. myeloid cell lineages [25]. 

## 4. Potential Influence of Host Protein Incorporation on HIV Pathogenesis

Upon incorporation into HIV virions, molecules can retain their biological activity and mediate some effects during the course of HIV infection. Depending on the nature of the host molecules incorporated, several potential impacts were proposed on HIV pathogenesis (e.g., HIV virions infectivity potential, cell attachment and immunoregulation) [12,17,19,29,30,60,61]. The following section will, therefore, underscore the major potential influence of host molecules incorporation on HIV pathogenesis. 

### 4.1. Potential Influence on Promoting HIV Virions Interaction with Susceptible Cells

**Figure 4:**
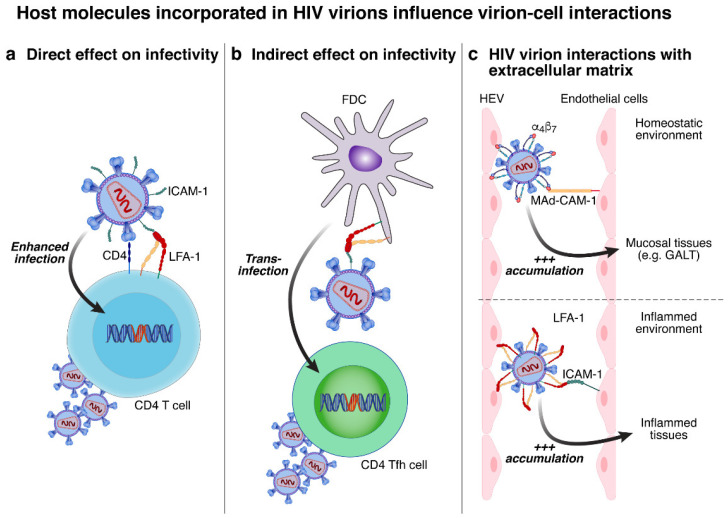
Host molecules incorporated into HIV virions influence virion-cell interactions. The incorporation of host molecules might influence HIV pathogenesis by promoting the interaction between HIV virions and susceptible cells. The incorporation of host molecules (e.g., ICAM-1) might promote HIV virion infectivity through: (**a**) the direct interaction with susceptible cells; and (**b**) trans-infection through the interaction with FDC. (**c**) Incorporated molecules (e.g., α4β7 and LFA-1) might promote HIV virion attachment to endothelial cells and thus enhancing the interaction with susceptible cells either in homeostatic or inflamed environments.

HIV virion interaction with the host cell is mediated by the viral glycoprotein gp120 interacting with the cellular receptor CD4, resulting in conformational changes in gp120 and allowing the interaction with one of the two co-receptors, CCR5 or CXCR4 [62]. However, the adhesion to the target cell can be reinforced by host proteins incorporated into the HIV virion lipid envelope, which can increase the binding affinity [30,63]. In this regard, certain incorporated molecules were proposed to play a role in enhacing HIV infectivity by promoting the direct interaction between HIV virions and susceptible cells (e.g., HLA-DR) (Figure 4a), while others may increase HIV infectivity through indirect interaction with follicular dendritic cells (FDCs) (e.g., ICAM-1) (Figure 4b), or with the extracellular matrix of high endothelial venules (HEVs) (e.g., α4β7 or LFA-1) (Figure 4c) [12,17,29,30].

ICAM-1: ICAM-1 is an adhesion molecule expressed mainly on endothelial cells and is playing a crucial role in the migration of immune cells expressing LFA-1 to inflammation sites [64]. Furthermore, the interaction between ICAM-1 and LFA-1 is also known to create an immunological synapse between dendritic cells and T cells thus promoting their interaction [65]. ICAM-1 incorporation into HIV virions was proposed to be mediated following interactions with Gag viral protein [13] and promote HIV virion infectivity by increasing virus attachment to susceptible LFA-1^+^ cells, thereby promoting the first steps of HIV replication cycle [17,66,67]. Indeed, no increase in infectivity was observed when LFA-1^−^ cells were infected with ICAM-1^+^ HIV virions, thus supporting these findings [68]. ICAM-1 incorporation might also promote HIV virions rolling on susceptible cells to maximize gp120-CD4 interaction, enhance the interaction with the co-receptor CXCR4, and induce cytoskeleton-remodeling [66,67,69]. Furthermore, ICAM-1 incorporation into HIV virions might also favor trans-infection from LFA-1^+^ cells (e.g., FDCs) to susceptible cells in LNs germinal centers (GC) [70,71].

α4β7: α4β7 is expressed on immune cells and has been described to play a fundamental role in T-cell homing towards the intestinal track through its interaction with MAdCAM-1 expressed on HEV in Peyer’s patches and the mesenteric lymph nodes [72]. A recent study based on SIV/SHIV-infected macaques demonstrated that the blockade of α4β7 reduced SIV/SHIV viral load both in plasma and gastrointestinal tissues and delayed the time to viral rebound after ART interruption [73,74]. The author subsequently proposed that α4β7 mAbs might have acted directly on α4β7^+^ HIV virions interrupting key events of viral infection [12]. Using an in vivo model, they showed that the incorporation of α4β7 into HIV virions might play a role in promoting HIV virions accumulation to MAdCAM-1^+^ on HEV [12]. Notably, a higher proportion of α4β7^+^ HIV virions was observed in plasma of viremic acute HIV-infected individuals compared to chronic HIV-infected individuals [12]. Based on these observations, a clinical trial using α4β7 mAbs in ART-treated HIV-infected individuals investigated the potential impact on HIV viral rebound. Unfortunately, this study did not achieve the same promising results as the one in macaques, and no delay in viral rebound was observed [75]. Therefore, the role of α4β7 mAbs in the therapeutic armamentarium for HIV cure remains debated.

Other adhesion molecules were shown to be incorporated into HIV virions (e.g., LFA-1 and CD62L) and it has been proposed that they play a role in enhancing HIV infectivity by promoting the interaction of HIV virions with susceptible cells through the interaction with their respective ligands (i.e., ICAM-1 and CD34/MAdCAM-1) in various settings (e.g., homeostatic vs. inflamed environment) [76,77,78,79].

### 4.2. Influence of T and B Cell Responses

**Figure 5:**
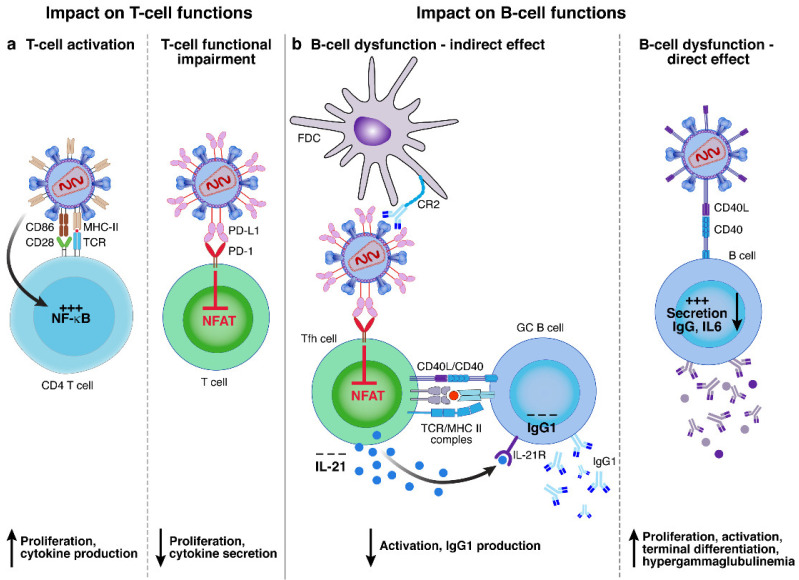
Potential influence of host protein incorporation on T and B cell responses. The incorporation of host molecules might influence HIV pathogenesis by modulating T and B cell responses: (**a**) the co-incorporation of HLA-DR and CD86 into HIV virions may favor T-cell activation by increasing NF-κB pathway. The incorporation of PD-L1 into HIV virions may favor T-cell functional impairment by inhibiting NFAT pathway; and (**b**) the incorporation of PD-L1 impacts B-cell functions through an indirect mechanism, by impairment of Tfh cell functions, which in turn may fail to provide efficient B-cell support. The impact of incorporated molecules on B-cell functions might involve direct or indirect interactions. The incorporation of CD40L into HIV virions interacts with CD40^+^ B cells and may promote B-cell activation.

Various incorporated molecules were proposed to impact immune cell function/activation (Figure 5), either by promoting B and/or T-cell activation (e.g., CD40L and HLA-DR alone or co-incorporated with CD86) or functional impairment (e.g., PD-L1) [18,19,60,61,80]. 

HLA-DR: HLA-DR is one of the MHC class II molecules expressed mostly on antigen-presenting cells (APCs) and is able to present peptides to CD4 T cells [81]. HLA-DR is also known to be upregulated on activated CD4 T cells [82]. HLA-DR was one of the first host molecules described to be incorporated into HIV virions. In addition to its role in increasing HIV infectivity through the enhancement of HIV virion fusion by the interaction of HLA-DR^+^ HIV virions with CD4, HLA-DR incorporation was proposed to influence T-cell function/activation [30,38,52,83]. Interestingly, it was also suggested that HLA-DR^+^ HIV virions may have the capacity to present peptide epitopes to CD4 T cells and may modulate CD4 T-cell responses (Figure 5a) [60,61,63]. The consequences of this stimulation may depend on the co-incorporation of the co-stimulatory molecule CD86 and the nature of the peptide presented [60,61]. Indeed, some authors proposed that HLA-DR^+^ HIV virions might be sufficient to trigger T-cell proliferation, cytokine production, and apoptosis in vitro, while other studies proposed that HLA-DR^+^ HIV virions might not trigger complete CD4 T-cell activation and thus may prime these cells towards anergy [60,61,63]. Another study proposed that HLA-DR^+^ HIV virions trigger T-cell apoptosis by increasing Fas and FasL expression levels [84].

PD-L1: The interaction between PD-1 on T cells and PD-L1 on APCs contribute to regulate T-cell proliferation and cytokines production and is proposed to play a major role in T-cell functional impairment [85,86]. It was demonstrated that PD-L1 was actively incorporated into HIV virions from plasma of viremic HIV-infected individuals with a process involving, at least in part, the interaction with HIV p17 matrix proteins. In this study, PD-L1^+^ HIV virions were able to significantly reduce T follicular helper (Tfh) cell proliferation and IL-21 production in vitro (Figure 5a), through the partial inhibition of T-cell signaling pathway [19]. Interestingly, the impact of PD-L1^+^ HIV virions on Tfh cell function ultimately translated into reduced IgG1 production from GC B cells in vitro (Figure 5b) [19]. Therefore, in this study, the authors showed that PD-L1^+^ HIV virions may not only influence directly CD4 T-cell functions but also indirectly influence B-cell responses. 

CD40L: CD40L is a co-stimularoy receptors expressed on T cells that play a fundamental role by interaction with CD40 on B cells during B-cell maturation and differentiation processes [87]. Several studies showed that CD40L^+^ HIV virions could interact with B cells and could induce unspecific B-cell proliferation, activation, and terminal differentiation as well as IgG production, and IL-6 secretion (Figure 5b) [18,80,88]. Interestingly, the quantity of IgG produced did not correlate with the presence or absence of Env but only depended on the presence of CD40L on HIV virion surface. The authors proposed that CD40L^+^ HIV virions might contribute to the hypergammaglobulinemia observed during the course of HIV infection [80,88]. At the intracellular level, CD40L^+^ HIV virions were able to induce an activation of the NF-κB pathway, which was similar to the one observed using CD40 antibody stimulation. Furthermore, CD40L^+^ HIV virions might also contribute to the increase the infection rate of CD4 T cells when co-cultured with tonsillar B cells, by promoting the secretion of pro-inflammatory cytokines by B cells, providing a favorable micro-environment for HIV replication [88]. 

## 5. Conclusions

During the budding process, HIV virions can passively and/or actively incorporate functionally active host molecules, which may play an active role during HIV pathogenesis. The development of novel experimental strategies and the advance of new technologies will soon uncover the complexity of this phenomenon, opening the way to a better understanding of HIV pathogenesis.

## Data Availability

Not applicable.
